# Supplementary material for: Precancerous niche remodelling dictates nascent tumour persistence
Source: Nature. 2026 Mar 4;653(8113):242–53. doi: 10.1038/s41586-026-10157-8 (PMC13148994; doi:10.1038/s41586-026-10157-8)
Supplement: Supplementary file 1 — Supplementary Methods [file 41586_2026_10157_MOESM1_ESM.pdf]

nature portfolio

https://doi.org/10.1038/s41586-026-10157-8

Supplementary information

Precancerous niche remodelling dictates nascent tumour persistence

In the format provided by the authors and unedited

Pre-cancerous Niche Remodelling Dictates Nascent Tumour Persistence

G. Skrupskelyte[1,2,#*], J. E. Rojo Arias[1,2,3#], H. Ajith[1,2], Y. Dang[4,5,6], D. Rossetti [1,2], S. Han[7], M. K. S. Tang[1,2], M. T. Bejar[1,2], B. Colom[8,9], J. C. Fowler[8], K. Murai[8], W. Knight[8], D. Aust[10,11,], M. H. H. Schmidt[12], J. Jászai[12], S. Zeki[13], A. Noorani[8,14], P. H. Jones[8,15], S. Rulands[5,16], B. D. Simons[1,7,17], M. P. Alcolea[1,2,*]

Affiliations:

[1]Cambridge Stem Cell Institute, Jeffrey Cheah Biomedical Centre, University of Cambridge, Puddicombe Way, Cambridge, CB2 0AW, UK.

[2]Department of Physiology, Development and Neuroscience, University of Cambridge, Cambridge CB2 3EG, UK.

[3]Currently at RhyGaze AG, Mittlere Strasse 91, 4031 Basel, Switzerland.

[4]Max Planck Institute for Molecular Cell Biology and Genetics, Pfotenhauerstrasse 108, 01307, Dresden, Germany

[5]Max Planck Institute for the Physics of Complex Systems, Noethnitzer Str. 38, 01187 Dresden, Germany

[6]Center for Systems Biology, Pfotenhauerstrasse 108, 01307, Dresden, Germany

[7]Gurdon Institute, University of Cambridge, Cambridge CB2 1QN, UK.

[8]Wellcome Sanger Institute, Hinxton CB10 1SA, UK

[9]Currently at Cambridge Institute of Science, Altos Labs, Cambridge CB21 6GP, UK

[10]Institut für Pathologie/Tumor and Normal Tissue Bank (TNTB), Universitätsklinikum Carl Gustav Carus, Technische Universität Dresden, Germany

[11]Institute of Pathology, University Hospital CGC Dresden, TU Dresden, Dresden, Germany

[12]Institut für Anatomie, Medizinische Fakultät Carl Gustav Carus, Technische Universität Dresden, Medizinische Fakultät, Fetscherstraße 74, 01307 Dresden, Germany

[13]Guy's and St Thomas' NHS Foundation Trust, London, UK

[14]Addenbrooke's Hospital, Cambridge University Hospital NHS Trust, Cambridge, UK.

[15]Department of Oncology, University of Cambridge, Hutchison Research Centre, Cambridge Biomedical Campus, Cambridge CB2 0XZ, UK

[16]Ludwigs-Maximilians-Universität München, Arnold Sommerfeld Center for Theoretical Physics, Theresienstr. 37, 80333 München, Germany

[17]Department of Applied Mathematics and Theoretical Physics, Centre for Mathematical Science, University of Cambridge, Cambridge, CB3 0WA, UK.

[#]These authors contributed equally to this work.

*Corresponding authors: gs463@cam.ac.uk (G.S.); mpa28@cam.ac.uk (M.P.A.).

Supplementary Information Guide

Supplementary Methods

Experimental mouse lines; strategy for imaging analysis and quantification of *in vitro* or *in vivo* experiments, as well as additional information on single-cell RNA sequencing sample preparation, processing, and transcriptional analysis. Main methods refer to relevant section in this supplement.

Supplementary Table 1.

Fibroblast lineage tracing coordinates (**Extended Data Fig. 4f, h, i**).

Supplementary Table 2.

Basic information for scRNA-seq sample collection. Quality control statistics for the pre and post processed scRNA-seq data (see Methods for filtering criteria). Cell numbers retained per condition, per cluster and per cell type after filtering.

Supplementary Table 3.

Cell types per cluster using multiple annotation methods (**Fig. 3b**; **Extended Data Fig. 5c-f**).

Supplementary Table 4

List of differentially expressed genes between assigned cell types.

Supplementary Table 5.

List of differentially expressed genes between fibroblast clusters in control conditions.

Supplementary Table 6.

List of differentially expressed genes between *Pdgfra*^{low} and *Pdgfra*^{high} fibroblast clusters split by conditions (**Extended Data Fig. 6a**).

Supplementary Table 7.

List of differentially expressed genes between cluster 19 and clusters 2 and 4 (**Extended Data Fig. 6e**).

Supplementary Table 8.

List of differentially expressed genes between conditions in each fibroblast cluster (**Extended Data Fig. 6c**).

Supplementary Table 9.

Genes commonly mutated in squamous cell carcinoma and used for targeted DNA sequencing of tumour stromal *versus* control tissue **(Extended Data Fig. 7a,b).**

Supplementary Table 10.

List of differentially expressed genes between immune cells (**Extended Data Fig. 7f-h**).

Supplementary Table 11.

List of differentially expressed genes identified by trajectory analysis. Genes are shown in groups according to the module to which they were assigned to (**Extended Data Fig. 8g).**

Supplementary Table 12.

Statistical analysis of gene modules identified in **Extended Data Fig. 8g.**

Supplementary Table 13.

List of differentially expressed genes between Tumour 12 and Tumour 1 populations (**Extended Data Fig. 8j**).

Supplementary Table 14

Pathway enrichment analysis of genes identified as differentially expressed in Tumour 12 relative to Tumour 1 keratinocytes **(Extended Data Fig. 8k).**

Supplementary Table 15.

List of antibodies used.

Supplementary Methods

Experimental mouse lines

To assess the proliferative state of cells, *$R26^{Fucci2aR}$* (*Fucci2a*) mice were used. This mouse line constitutively expresses fluorescent reporters that highlight different phases of the cell cycle (G1 is marked by *mCherry-hCdt1*, and S/G2/M by *mVenus-hGem*). To identify fibroblasts, *$Pdgfra^{EGFP}$* mice were used. These mice constitutively express an H2B-eGFP fusion peptide from the endogenous *Pdgfra* locus. For *Sox9* conditional knock-out experiments, *$Sox9^{flox/flox}$* were crossed with *$K14$-Cre^{ER}* mice to generate *$K14^{CreER}$/ $Sox9^{flox/flox}$* animals in which *Sox9* expression is inactivated in recombined epithelial cells upon tamoxifen (TAM) administration. To visualise cells in 3D cultures (epithelioid)[57] cells *in vitro* the fluorescent reporter mouse line *$R26^{mT\text{-}mG}$* (*mTmG*), which constitutively express tdTomato localised in the cell membrane, was crossed with *$K14^{CreER}$/$Sox9^{flox/flox}$* and the conditional knock out phenotype was visualised *in vitro*. For fibroblast lineage tracing, *$Col1a2^{CreER}$* or *$Pdgfra^{CreERT}$* mice expressing a Tamoxifen-inducible *Cre* recombinase under the control of the endogenous *Col1a2* or *Pdgfra* promoter were used. This line was crossed with *$R26^{FlConfetti}$* mice in which the Rosa26 locus was targeted with a transgenic cassette containing the sequences for stochastic expression of either cyan, green, yellow or red fluorescent protein (CFP, GFP, YFP and RFP) upon *Cre* recombination. Thus, by using low doses of TAM in *$Col1a2^{CreER}$/$R26^{FlConfetit/WT}$*, individual Col1a2-expressing cells and their progeny can be labelled with one of these four fluorescent reporters. To identify the origin of tissues in 3D tissue recombination culture assays (donor or recipient), *$R26^{nT\text{-}nG}$* (*nTnG*) and *H2B-EGFP* (*CAG::H2B-EGFP*) mice were used. *$R26^{nT\text{-}nG}$* ubiquitously express a *tdTomato* fluorescent reporter that localises to the nucleus, while *H2B-EGFP* mice constitutively express EGFP that localises to the nucleus. In transplantation assays, mice commonly known as NOD scid gamma (NSG; NOD.Cg-*$Prkdc^{scid}$ $Il2rg^{tm1Wjl}$*/SzJ) were used as recipients. Due to the absence of *Prkdc* and X-linked *Il2rg* expression, these mice are immunodeficient.

Analysis of migration assay

The total number of fibroblast and the number of fibroblasts that crossed the transwell insert membrane were counted and migration rates calculated as the fraction of migrated fibroblasts relative to all fibroblasts detected. Membranes from transwell inserts were visualised by their autofluorescence after excitation with the 405 laser. Displayed images were produced using Volocity 5.5.5.

Assessment of tumour phenotype, size and number in whole-mounts

To identify tumours in the upper gastrointestinal tract as early as 10 days after DEN withdrawal, we used DAPI to assess changes in tissue topology, as well as cell morphology. This was combined with immunostaining against Keratin 6A (KRT6A) - a tumour marking keratin[1]. PDGFRα was used as a marker of residential fibroblasts to assess niche phenotype. Approximately one third of the oesophagus (middle part) was imaged using the following settings: a 40× objective, zoom 0.75, an optimal pinhole size (as defined by the software), a scan speed of 400 Hz, a line average of 1, a Z-step size of 1.5 [illegible]m, and a resolution of 512 × 512 pixels. Tumour phenotype (Niche- or Niche+) was visually scored by assessing basal keratinocyte morphology, the level of fibroblast recruitment, and fibroblast remodelling in a single-tile section view using Volocity 5.5.5 or 7. For clarity, exposure settings were adjusted to PDGFRα^{low} population when images were focussed on lamina propria alone and adjusted accordingly when both lamina propria and submucosa (PDGFRα^{high}) populations were displayed. Tumour diameter was scored in Volocity by measuring the distance between the first and last tumour nuclei along the transversal tumour axis in one plane. The number of tumours detected per tissue was normalised to the surface area analysed (typically 10-50 mm^2). To estimate the number of tumours per oesophagus, the surface areas of 4 whole oesophagi were measured and the total number of tumours extrapolated accordingly.

Proliferation, SOX9 and stromal cell distribution measurements in whole-mounts

EdU$^+$ and SOX9$^+$ tumour keratinocytes were quantified by manually cropping the stromal part of 3D images and running an automated pipeline for nuclear signal detection in Volocity 5.5.5. Control measurements were created by cropping regions of equivalent size in control areas. The number of identified objects or the sum of mean object signal intensities were normalised to surface area of each tumour, which was calculated by using the formula $S= \pi ab$ (a, width; b, height). The latter assumes that, in a single XY plane, the shape of an oesophageal tumour is an ellipse.

SOX9 mean intensities per object were summed per area of interest and normalised to the surface area of interest as well as to the individual sample average (self-average) to account for differences in intensity across imaging sessions.

Stromal cell distribution in Niche+ and Niche- tumours was estimated by counting all stromal cells in the lamina propria directly underneath the tumour epithelium and subsequently normalising this number to the tumour XY cross-sectional area. Cells were then identified as

fibroblasts (PDGFRα$^+$), immune (CD45$^+$) or endothelial (CD31$^+$) and their numbers expressed as a percentage of total stromal cells.

Fibroblast proliferation in tumours was scored by manually counting Ki67$^+$ EGFP$^+$ cells in *Pdgfra*^{EGFP} DEN-treated mice. The number of proliferating fibroblasts was normalised to the total number of fibroblasts in the lamina propria directly underneath the epithelial tumour.

Fibroblast density and proximity to SOX9$^+$ keratinocytes

Oesophageal tissues collected 10 days after DEN withdrawal were used to source SOX9$^+$ keratinocytes outside the tumour area. These were KRT6A-expressing, SOX9-expressing cell clusters (between 5-28 cells in size) that had normal topology as assessed by the position of DAPI-labelled nuclei. Tissues were additionally immunostained with antibodies against β-catenin to identify keratinocytes and against PDGFRα to identify fibroblasts. The number of fibroblasts in the lamina propria region directly underneath SOX9$^+$ keratinocyte clusters was counted manually and normalised to the surface area considered; equivalently sized areas in tissues of untreated animals were used as controls. The distance between SOX9-labelled keratinocyte clusters and the 5 closest fibroblasts was manually measured in either an xz or an yz cross-section view using Volocity 5.5.5 and is expressed as an inverse distance to demonstrate proximity.

Fibroblast lineage tracing

Oesophagi from *Col1a2*$^{CreER/WT}$ *R26*$^{FlConfetti/WT}$ mice that received TAM at the beginning of the DEN treatment were collected 6 months after DEN withdrawal. Tissues were processed and stained for PDGFRα and DAPI as described above. The more abundant YFP$^+$ and RFP$^+$ clones within tumours and in normal tissue regions were imaged on a confocal microscope and analysed with Volocity 5.5.5. To investigate clonal expansion of YFP$^+$ and RFP$^+$ fibroblasts, the coordinates of the tumour lobe (defined as the region between its basal keratinocyte layer and the adjacent lamina propria) were recorded in XY at intervals of 1-2 µm; the process was repeated at different Z positions throughout the entirety of the tumour (every 1-4 µm). Similarly, the XYZ coordinates of the nucleus of each confetti-labelled fibroblast found within the field of view as well as its colour were recorded. The average distance to the nearest fibroblast neighbour of the same colour was calculated and normalised to the distance to the nearest fibroblast neighbour of a different colour. Statistical significance was assessed using the Mann-Whitney U test (Wilcoxon signed-rank test).

Second-harmonic generation (SHG) imaging and analysis

Extracellular matrix (ECM) fibres in whole-mounts were imaged by SHG microscopy in a Zeiss LSM880 microscope. Prior to imaging, fixed oesophagi were incubated in PBS with 10 drops of NucRed647 (nuclear staining; Life Technologies, R37106) for 1 h at RT to label cell nuclei. Immediately after, samples were mounted using RapiClear 1.52 medium. For image acquisition, a Plan-Apochromat 40×/1.3 Oil DIC M27 objective was used. Tumours were identified by their characteristic morphology using a 633 nm laser to excite the NucRed647 nuclear dye, detecting its emission with a 638-704 nm filter. After the region of interest was defined, the tuneable laser of the instrument was set to 1040 nm (gain set to 780) to generate second-harmonic light from the ECM fibres in the tissue, which was detected with a 481-526 nm filter. Nuclei and ECM fibres were each imaged independently for every region of interest and merged afterwards for visualisation using the ZEN software (Blue edition, version 3.1, Carl Zeiss Microscopy GmbH). The microscope pinhole was set to its maximal aperture, namely 600 µm, and 2× line averaging was used. Images were acquired every 1 µm along the Z axis with an XY resolution of 1024 x 1024 pixels, a depth of 12 bits per pixel, and a pixel dwelling time of 0.77 µs.

Using the Fiji (2.0.0) software for bioimage analysis, a region of 200x200 pixels was arbitrarily selected within the lamina propria in each control image. In the case of tumour images, such region was selected to be directly underneath the lesion site. Fibre alignment was measured within these regions using the OrientationJ plug-in for Fiji[74] with the σ tensor value set to 7. For determining the emission intensity of extracellular matrix fibrils, images of the lamina propria were first exported in the TIFF format using ZEN. Within each control image, intensity was measured in four equivalent circular regions (phantom tumours; diameter = 125 pixels) and averaged. Only one such region immediately under the lesion site was considered for tumour images.

Transcriptomics

Single-cell and RNA isolation for single-cell RNA-Sequencing (scRNA-Seq)

For scRNA-Seq, *Fucci2a* male mice were sacrificed 8 months after DEN treatment (age-matched vehicle treated mice were used as controls). After dissection, as described above, tissues were flattened with the epithelial side up and visible tumour lesions were marked by a partial incision using a punch biopsy tool (1 mm diameter) under the dissecting microscope. Then, oesophagi and forestomachs were cut in half and incubated in 50 mM EDTA in PBS containing 1 U/µl RNAse Inhibitor (Life Technologies; AM2696) at 37°C for 30 minutes. Following incubation, the

epithelium was carefully peeled away from the stroma using fine forceps. Epithelium and stroma were each flattened, and tumour areas fully excised using 0.5-2 mm punch biopsies (to fit tumour size). Epithelium and stroma biopsies were kept separated. Biopsies were pooled from two to four animals (**Supplementary Table 2**). Stroma was thoroughly minced and incubated in 4 mg/ml collagenase type 4 (Thermo Fisher Scientific; 17104019) in DMEM with 1 U/ml RNAse inhibitor for 1 h at 37°C in a rotator. EDTA was added to the samples at a final concentration of 5 mM, and the suspension was diluted 1:5 in collection buffer (2 % heat-inactivated fetal bovine serum, Life Technologies; 26140079; 25 mM HEPES, Life Technologies; 15630056) in PBS to reduce the collagenase activity. Cell suspensions were filtered through a 100 µm cell strainer, centrifuged at 300×g for 5 min at 4°C, and resuspended in collection buffer before an additional filtering step with a 40 µm FlowMi tip filter (Thermo Fisher Scientific; 15342931) was performed to obtain single-cell suspensions. Cells were centrifuged at 300×g for 5 min at 4°C and resuspended in collection buffer containing 1U/µl RNAse. In parallel, the epithelium was thoroughly minced and incubated for 10 min at 37°C in 0.5mg/ml dispase (Sigma-Aldrich; D4818) in PBS containing 1 U/µl RNAse to create an epithelial cell suspension. EDTA was then added to the samples at a final concentration of 5mM and the suspension diluted 1:5 in collection buffer to reduce dispase activity. A single-cell suspension was obtained by filtering the samples through a 30 µm cell strainer. Cells were centrifuged at 300×g for 10 min at 4°C and resuspended in collection buffer containing 1 U/µl RNAse. Epithelial and stromal cells were then pooled together by condition (Control, Ctrl; Adjacent non-tumour epithelium, DEN; and Tumour) prior to proceeding to library production for scRNA-seq.

Data clustering, annotation and enrichment

The shared nearest-neighbour (SNN) graph constructed with the FindNeighbours function was clustered using FindClusters with the Leiden community detection algorithm, resulting in 22 clusters (**Extended Data Fig. 5c**). Automated cell-type annotation was performed initially with Celltypist (version 1.6.3), a logistic regression classifier using a Gut cell atlas[75]. We then used a label transfer approach with FindTransferAnchors and TransferData functions of Seurat, referencing datasets from Grommisch et al. (2024)[73] and Yao et al. (2020)[41] (**Extended Data Fig. 5d; Supplementary Table 3**) for finer cell type granularity. These automated annotations were used to support manual annotation based on canonical marker genes (**Extended Data Fig. 5e,f**; **Supplementary Table 3**), resulting in the classification of cells into 11 major groups. Within the dataset, we defined three experimental conditions: age-matched controls (**Ctrl**), DEN-treated but histologically normal tissue (**DEN**), and **tumours** from DEN-treated mice. No clear separation between Ctrl, DEN, and tumour conditions was observed (**Extended Data Fig. 5g**). Cell type

distributions across conditions were compared using a Chi-squared test of independence. A contingency table of cell type counts by condition was constructed, and Chi-squared standardised residuals were calculated to assess the representation of specific cell types within each condition. Cell type proportions were computed per condition and visualised as stacked barplots. Standardised residuals were displayed as heatmaps indicating relative depletion or enrichment of each cell type in each condition.

Cell transition trajectory analysis

Basal epithelial cells were subset to create a new Seurat object, which was pre-processed with a similar approach as described above. The resulting clustered and dimensionally reduced object was converted into a cell_data_set using the as_cell_data_set function from SeuratWrappers (v0.4.0) and subsequently analysed in Monocle3 (v1.4.26)[76]. Trajectories were inferred with the learn_graph function, and cells were ordered along pseudotime using order_cells. Cluster C3, identified as a basal cell cluster containing a mix of conditions (**Extended Data Fig. 9a–d**), was selected as the root; this choice was further supported by RNA velocity analysis with scVelo (version 0.3.2)[77] using spliced and unspliced counts generated by velocyto[78] (version 0.17.17) (**Extended Data Fig. 9e**). The inferred trajectory resolved into two main branches, designated as the Tumour branch (comprising clusters C3, 1, 0, 8, 9, 2, and 6) and the Basal branch (clusters C3, 1, 7, 5, and 4). For each branch, cells were grouped into 100 pseudotime bins, and pseudotime values were normalised to range from 0 to 1 to allow direct comparison between branches. Genes associated with pseudotime progression were identified with the graph_test function from monocle3 ($q < 0.05$), and the resulting top 1,500 genes ranked by Moran's I statistic were smoothed along pseudotime within each branch using locally estimated scatterplot smoothing (loess). Smoothed expression matrices were scaled, ordered by branch-specific pseudotime, and visualised as heatmaps using the ComplexHeatmap package (version 2.22.0). Heatmaps were further organised by applying k-means row splitting to partition genes into 15 co-expression modules. Module dynamics were then summarised by computing the average expression of all genes within a module across pseudotime bins and visualised as line plots of average module expression versus normalised pseudotime. For comparability across modules, y-axis limits were fixed globally based on the observed range of expression values. Modules enriched along the **Tumour branch** were identified by comparing the difference in mean expression between the first and last 10% of pseudotime bins (Δ) (**Supplementary Table 12**). Modules with $\Delta\_tumour > 2$ (showing strong upregulation towards the end of the tumour trajectory) and $\Delta\_basal < 1$ (indicating minimal change along the Basal branch), Specifically Module 1 and 12 were retained for downstream analysis, ensuring that

selected modules were enriched in the tumour trajectory while largely invariant in basal cells. To identify cells enriched for these programmes, we applied AUCell (v1.28.0) to the raw count matrix from basal epithelial cells, ranking cells by their expression of Module 1 and Module 12 gene sets. Cells above the 80th percentile of the corresponding AUC distributions were selected, with Module 1 cells restricted to those not overlapping with Module 12. Populations assigned from the modules were then visualised on UMAP embeddings. Differential expression between the resulting populations was performed using Seurat's FindMarkers function ($log_2FC > 0.5$, adjusted $p < 0.05$) and visualised using the EnhancedVolcano (version 1.24.0) package.

Analysis of interactions between cell types by CellChat

To quantitatively analyse cell-to-cell communication networks from scRNA-seq data, we used CellChat (version 2.2.0), a tool that infers ligand-receptor interactions (http://www.cellchat.org/). To analyse the interactions within different immune cell types, we identified cell types that are enriched in Tumour cells, and selected 8 such cell types (**Fig. 5a,b**). For interactions between keratinocytes and fibroblasts, we used Tumour enriched C19 Fibroblasts and previously identified tumour enriched Module 12 and 1 populations along with non-tumour, internal controls. Using these groups as input, the significant ligand-receptor interactions were identified and quantified based on CellChat's function for computing communication probabilities for overexpressed ligand-receptor pairs using default parameter settings (trimean expression value for each group, p-value < 0.05). Total interaction strengths represent the sum of all incoming and outgoing communication probabilities for each cell cluster (and for each given pathway, if applicable). Scores for sender and receiver roles of cell clusters were computed from network centrality scores implemented through CellChat's netAnalysis_computeCentrality function.

Differential gene expression and gene set enrichment analysis (GSEA)

Gene Ontology analysis was performed using with g:profiler (https://biit.cs.ut.ee/gprofiler/gost version e109_eg56_p17_1d3191d) and STRING (https://string-db.org/ version 12.0). Gene Ontology biological processes (GO:BP) were grouped to mother terms in Revigo (http://revigo.irb.hr/ version 1.8.1). The resulting GO:BP mother terms, Kyoto Encyclopaedia of Genes and Genomes (KEGG) (https://doi.org/10.1093/nar/28.1.27) terms and Reactome (REAC) (https://doi.org/10.1093/database/baz123) terms were manually grouped, with the most representative significant terms selected for depiction based on experimental observations and biological knowledge (see **Supplementary tables 14**).

Fibroblasts expressing *Pdgfra* were categorised as either $Pdgfra^{low}$ (clusters2, 4 and 19) or $Pdgfra^{high}$ (cluster 3) and genes differentially expressed between them were identified using the FindMarkers function of the Seurat package with standard parameters. The $\log_2$ fold-change of significantly regulated genes encoding proteins contained in the matrisome reference list[34] was plotted as a heatmap using GraphPad Prism.

Similarly, in cluster 19, DEGs between conditions (namely, Tumour vs control and Tumour vs DEN) were identified using the FindMarkers function of the Seurat package. DEGs with a p-value < 0.05 and a $\log_2$ fold-change above 0.5 were cross-referenced with known cancer-associated fibroblast (CAF) markers. The overlap is schematically shown in a Venn diagram (generated in the website https://www.bioinformatics.org/gvenn/)[37,79].

Immune cells were further annotated using a label transfer approach with the single-cell reference dataset from Yao et al. 2020[41] to achieve finer granularity. Annotation accuracy was evaluated by generating a heatmap of curated marker genes (from Yao et al. 2020), where aggregated and scaled expression profiles were compared across the assigned cell types (Extended Data Figure 8c). Marker genes for each immune cell type were subsequently identified using Seurat's FindAllMarkers function (Supplementary Table 10) with p-value < 0.05 and a $\log_2$ fold-change above 0.5.

74 Rezakhaniha, R. *et al.* Experimental investigation of collagen waviness and orientation in the arterial adventitia using confocal laser scanning microscopy. *Biomech Model Mechanobiol* **11**, 461–473 (2012). https://doi.org/10.1007/s10237-011-0325-z

75 Oliver, A. J. *et al.* Single-cell integration reveals metaplasia in inflammatory gut diseases. *Nature* **635**, 699–707 (2024). https://doi.org/10.1038/s41586-024-07571-1

76 Cao, J. *et al.* The single-cell transcriptional landscape of mammalian organogenesis. *Nature* **566**, 496–502 (2019). https://doi.org/10.1038/s41586-019-0969-x

77 Bergen, V., Lange, M., Peidli, S., Wolf, F. A. & Theis, F. J. Generalizing RNA velocity to transient cell states through dynamical modeling. *Nat Biotechnol* **38**, 1408–1414 (2020). https://doi.org/10.1038/s41587-020-0591-3

78 La Manno, G. *et al.* RNA velocity of single cells. *Nature* **560**, 494–498 (2018). https://doi.org/10.1038/s41586-018-0414-6

79 Yang, D., Liu, J., Qian, H. & Zhuang, Q. Cancer-associated fibroblasts: from basic science to anticancer therapy. *Exp Mol Med* **55**, 1322–1332 (2023). https://doi.org/10.1038/s12276-023-01013-0
