# Supplementary material for: Precancerous niche remodelling dictates nascent tumour persistence
Source: Nature. 2026 Mar 4;653(8113):242–53. doi: 10.1038/s41586-026-10157-8 (PMC13148994; doi:10.1038/s41586-026-10157-8)
Supplement: Supplementary file 2 — Reporting Summary [file 41586_2026_10157_MOESM2_ESM.pdf]

nature portfolio

Corresponding author(s): Maria P. Alcolea and Greta Skrupskelyte

Last updated by author(s): 09/12/2025

Reporting Summary

Nature Portfolio wishes to improve the reproducibility of the work that we publish. This form provides structure for consistency and transparency in reporting. For further information on Nature Portfolio policies, see our Editorial Policies and the Editorial Policy Checklist.

Statistics

For all statistical analyses, confirm that the following items are present in the figure legend, table legend, main text, or Methods section.

n/a	Confirmed	
☐	☒	The exact sample size (*n*) for each experimental group/condition, given as a discrete number and unit of measurement
☐	☒	A statement on whether measurements were taken from distinct samples or whether the same sample was measured repeatedly
☐	☒	The statistical test(s) used AND whether they are one- or two-sided<br>*Only common tests should be described solely by name; describe more complex techniques in the Methods section.*
☐	☒	A description of all covariates tested
☐	☒	A description of any assumptions or corrections, such as tests of normality and adjustment for multiple comparisons
☐	☒	A full description of the statistical parameters including central tendency (e.g. means) or other basic estimates (e.g. regression coefficient) AND variation (e.g. standard deviation) or associated estimates of uncertainty (e.g. confidence intervals)
☐	☒	For null hypothesis testing, the test statistic (e.g. *F*, *t*, *r*) with confidence intervals, effect sizes, degrees of freedom and *P* value noted<br>*Give P values as exact values whenever suitable.*
☒	☐	For Bayesian analysis, information on the choice of priors and Markov chain Monte Carlo settings
☐	☒	For hierarchical and complex designs, identification of the appropriate level for tests and full reporting of outcomes
☒	☐	Estimates of effect sizes (e.g. Cohen's *d*, Pearson's *r*), indicating how they were calculated

Our web collection on statistics for biologists contains articles on many of the points above.

Software and code

Policy information about availability of computer code

Data collection

LAS X 4.7.0.28176 and 3.5.5.19976
Volocity 5.5.5, PerkinElmer
Volocity 7, Quorum technologies
Zen 3.2, Zeiss
Arivis 3.5.1, Zeiss
Fiji (2.0.0)

Data analysis

Confocal images were obtained and analysed using LAS X 4.7.0.28176 and 3.5.5.19976 and Zen3.2, Zeiss softwares. Further image analysis was performed using Volocity 5.5.5, PerkinElmer; Volocity 7, Quorum technologies; Arivis 3.5.1, Zeiss and Imaris Viewer 10.1.1
Cellranger (v7.0.1);

Mapping scRNAseq data to reference genomeSeurat (v5.0.3); Processing counts matrix and downstream analysisScrublet (v0.2.3); Doublet removalHarmony (v1.2.3); Integration and batch effect correctionSeuratWrappers (v0.4.0); Used to convert seurat object to cell_data_set (monocle 3)Monocle3 (v1.4.6); Pseudotime analysisCellChat (v2.2.0); Cell-Cell Communication analysesEnhancedVolcano (v1.24.0); Volcano PlotsGgplot2 (v3.5.2); PlotsGSEABase (v1.68.0); Gene list format correction for AUCellAUCell (v1.28.0); Scoring cells based on gene setsCowplot (v1.2.0); PlotsComplexHeatmaps (v2.22.0): Heatmaps.

The analysis code is available on the AlcoleaLab GitHub page https://doi.org/10.5281/zenodo.17802564

DNA sequencing analysis was performed using BWA-mem (0.7.17); SAMtools (v1.11); deepSNV R package (ShearwaterML; version 1.21.3); VAGrENT (3.3.0).

Statistical analysis was performed using GraphPad Prism (10.5.0)

For manuscripts utilizing custom algorithms or software that are central to the research but not yet described in published literature, software must be made available to editors and reviewers. We strongly encourage code deposition in a community repository (e.g. GitHub). See the Nature Portfolio guidelines for submitting code & software for further information.

Data

Policy information about availability of data

All manuscripts must include a data availability statement. This statement should provide the following information, where applicable:

- Accession codes, unique identifiers, or web links for publicly available datasets
- A description of any restrictions on data availability
- For clinical datasets or third party data, please ensure that the statement adheres to our policy

Mouse reference genomes GRCm38 and mm10 2020-A were used. The single cell RNA sequencing data generated in this study have been deposited in the Gene Expression Omnibus (GEO) repository under accession code GSE271962. The DNA sequencing dataset was deposited at the European nucleotide Archive (ENA) under dataset accession number ERP134942. Source data are provided with this paper.
The analysis code is available on the AlcoleaLab GitHub page https://doi.org/10.5281/zenodo.17802564

Research involving human participants, their data, or biological material

Policy information about studies with human participants or human data. See also policy information about sex, gender (identity/presentation), and sexual orientation and race, ethnicity and racism.

Reporting on sex and gender	Sex and gender were not considered in this study.
Reporting on race, ethnicity, or other socially relevant groupings	Race, ethnicity or other social group was not considered in this study.
Population characteristics	To enable the recruitment of as many as possible early stage squamous cell carcinoma tumour samples from patients >18 years old were recruited from tissue bank.<br>Shortly, the T1A and T1B stage chemo-naive surgical tumour samples were donated by patients who had undergone surgery at the Clinic for Visceral, Thoracic and Vascular Surgery at TU Dresden or at the Medical Department I, of the Carl Gustav Carus University Hospital. The corresponding FFPE material (tumour and normal tissue) from a total of ten characterised oesophageal squamous cell carcinomas was selected from the archive of the Institute of Pathology of the University Hospital Carl Gustav Carus (EK 59032007) by the Tumour and Normal Tissue Bank (TNTB) Dresden.<br>Tis and ypT0 (prior to chemo T2N0M0) stage surgical tumour samples were donated by patients who had undergone surgery at Guy's and St Thomas' (London, UK) and Addenbrooke's Hospitals (Cambridge, UK), respectively.
Recruitment	Patient information sheet and signed consent were used to recruit prospective cases. Prospective samples for this study were collected and processed in accordance with local and national regulations with written consent and transferred using methods fully compliant with the requirements of the Human Tissue Act and the Human Tissue Authority.
Ethics oversight	The T1A and T1B stage chemo-naive surgical tumour samples were donated by patients who had undergone surgery at the Clinic for Visceral, Thoracic and Vascular Surgery at TU Dresden or at the Medical Department I, of the Carl Gustav Carus University Hospital. Macroscopically normal samples adjacent to the proximal resection margin were sampled from cancer resection specimens. The corresponding FFPE material (tumour and normal tissue) from a total of ten characterised oesophageal squamous cell carcinomas was selected from the archive of the Institute of Pathology of the University Hospital Carl Gustav Carus (EK 59032007) by the Tumour and Normal Tissue Bank (TNTB) Dresden. Studies presented in the manuscript involving early chemo-naïve human oesophageal tumour samples from Dresden were approved by the Ethics Committee of TU Dresden, Germany (Ref.: SR+BO-ff (Mono)-EK-161042025). Studies presenting chemo-naïve or post-chemo human oesophageal tumour samples from Guy's and St Thomas' (London, UK) and Addenbrooke's Hospital (Cambridge, UK), respectively were approved by East of Scotland Research Ethics approval committee (REC 18/ES/133).

Note that full information on the approval of the study protocol must also be provided in the manuscript.

Field-specific reporting

Please select the one below that is the best fit for your research. If you are not sure, read the appropriate sections before making your selection.

☒ Life sciences ☐ Behavioural & social sciences ☐ Ecological, evolutionary & environmental sciences

For a reference copy of the document with all sections, see nature.com/documents/nr-reporting-summary-flat.pdf

Life sciences study design

All studies must disclose on these points even when the disclosure is negative.

Sample size	Sample size was determined from pilot studies and previous published research studies as listed below:<br>Colom, B. et al. Mutant clones in normal epithelium outcompete and eliminate emerging tumours. Nature 598, 510-514 (2021). https://doi.org:10.1038/s41586-021-03965-7<br><br>Frede, J., Greulich, P., Nagy, T., Simons, B. D. & Jones, P. H. A single dividing cell population with imbalanced fate drives oesophageal tumour growth. Nat Cell Biol 18, 967-978 (2016). https://doi.org:10.1038/ncb3400<br><br>A minimum of three independent mice or ex vivo cultures were used in all cases. For image analysis, a minimum of three independent samples were inspected and replicate images were taken per sample for data to be analysed both internally within said sample, and between at least 3 biological replicates.
Data exclusions	Exclusions were made to the data in the case of single-cell RNA sequencing analysis to exclude low quality cells as is considered best practice in such analyses. In this case, doublets were identified and removed using Scrublet, with per-sample doublet score thresholds between 0.1 and 0.2. Cells were also filtered on numbers of detected genes (nFeature_RNA) or total transcript counts (nCount_RNA) on a per-sample basis, with lower and upper limits ranging from 700–1,500 genes to 8000-10000 genes and 2,000–4,000 to 40000-160,000 transcripts per cell, respectively. Cells with more than 15% of reads mapping to mitochondrial genes were also excluded. These thresholds were determined individually for each library to account for differences in library quality.
Replication	A minimum of three independent mice or ex vivo cultures were used in all cases. All experiments were performed independently at least three times with similar results, unless otherwise stated. The reproducibility of all key findings was confirmed in at least three independent experiments conducted on different days and using independent biological samples.
Randomization	Mice and ex vivo cultures were randomly assigned to experimental groups.
Blinding	Blinding was performed for tumour count per condition and in vitro sample analyses by confocal microscopy. In cases where quantification was performed in tumours and the morphologically normal areas blinding was not possible due to differences in physical sample appearance. In ex vivo cultures where perturbation assay resulted in inhibition of fibroblast to keratinocyte interaction blinding was not possible due to differences in sample appearance.

Reporting for specific materials, systems and methods

We require information from authors about some types of materials, experimental systems and methods used in many studies. Here, indicate whether each material, system or method listed is relevant to your study. If you are not sure if a list item applies to your research, read the appropriate section before selecting a response.

Materials & experimental systems

n/a	Involved in the study	
☐	☒	Antibodies
☒	☐	Eukaryotic cell lines
☒	☐	Palaeontology and archaeology
☐	☒	Animals and other organisms
☒	☐	Clinical data
☒	☐	Dual use research of concern
☒	☐	Plants

Methods

n/a	Involved in the study	
☒	☐	ChIP-seq
☒	☐	Flow cytometry
☒	☐	MRI-based neuroimaging

Antibodies

Antibodies used	Primary antibodies<br>AREG; R&D Systems, Inc; AF989<br>AREG; R&D Systems, Inc; AF262<br>αSMA; Abcam; ab5694<br>β-Catenin; Cell Signaling Technology; 9562<br>CD31; Abcam; ab7388<br>CD45; BioLegend; 103102<br>FAP; Abcam; ab218164<br>FINC; BD Biosciences; 610078<br>K17; Proteintech; CL488-17516<br>KRT6A; BioLegend; 905701<br>KRT6A; Abcam; ab18586<br>KI67; Abcam; ab16667<br>PDGFRα; R&D Systems, Inc; AF1062<br>PDGFRα; R&D Systems, Inc; AF307

S100A4 (FSP); Fisher Scientific Ltd; PA5-16586
SOX9; Millipore; AB5535
VIM; Abcam; ab194719
active YAP; Abcam; ab205270
EGR1, Abcam; ab300449
RUNX1, Proteintech; 25315-1-AP
Secondary antibodies
Goat IgG 647; Millipore; AP180SA6
Goat IgG 750; Abcam; ab175745
Mouse IgG 488; Invitrogen; A-21202
Mouse IgG 647; Invitrogen; A-31571
Mouse IgG 750; Abcam; ab175738
Rabbit IgG 488; Invitrogen; A-21206
Rabbit IgG 555; Invitrogen; A-31572
Rabbit IgG 647; Invitrogen; A-31573
Rabbit IgG 750; Abcam; ab175728
Rabbit IgG; Abcam; ab171870
Rat IgG 647; Abcam; ab150155
Rat IgG 750; Abcam; ab175750

Validation

Well characterised antibodies were used in this study. All are commercially available and validated by the manufacturers. List below target molecule, host, company link

AREG, goat, https://www.rndsystems.com/products/mouse-amphiregulin-antibody_af989
AREG, goat https://www.rndsystems.com/products/human-amphiregulin-antibody-31221_mab262
A-SMA, rabbit, https://www.abcam.com/en-gb/products/primary-antibodies/alpha-smooth-muscle-actin-antibody-ab5694
β-Catenin, rabbit, https://www.cellsignal.com/products/primary-antibodies/b-catenin-antibody/9562
CD31, rat, https://www.abcam.com/en-gb/products/primary-antibodies/cd31-antibody-mec-746-ab7388
CD45, rat, https://www.biolegend.com/en-gb/products/purified-anti-mouse-cd45-antibody-102
FAP, rabbit, https://www.abcam.com/en-gb/products/primary-antibodies/fibroblast-activation-protein-alpha-antibody-ab218164
Fibronectin, mouse, https://www.bdbiosciences.com/en-gb/products/reagents/flow-cytometry-reagents/research-reagents/single-color-antibodies-ruo/purified-mouse-anti-fibronectin.610077
K17, rabbit, https://www.ptglab.com/products/Cytokeratin-17-Specific-Antibody-CL488-17516.htm
KRT6A, rabbit, https://www.biolegend.com/en-gb/products/purified-anti-mouse-keratin-6a-antibody-11459
KRT6A, mouse, https://www.abcam.com/en-us/products/primary-antibodies/cytokeratin-6-antibody-ks6ka12-ab18586
KI67, rabbit, https://www.abcam.com/en-gb/products/primary-antibodies/ki67-antibody-sp6-ab16667
PDGFRα, goat, https://www.rndsystems.com/products/mouse-pdgf-ralpha-antibody_af1062
PDGFRα, goat https://www.rndsystems.com/products/human-pdgf-ralpha-antibody_af-307-na
S100A4 (FSP); rabbit, https://documents.thermofisher.com/TFS-Assets/LSG/certificate/Certificates-of-Analysis/PA516586_SK2481855D.PDF
SOX9, rabbit, https://www.merckmillipore.com/GB/en/product/Anti-Sox9-Antibody,MM_NF-AB5535
VIM, rabbit, https://www.abcam.com/en-do/products/primary-antibodies/alexa-fluor-647-vimentin-antibody-epr3776-cytoskeleton-marker-ab194719
active YAP, rabbit, https://www.abcam.com/en-gb/products/primary-antibodies/active-yap1-antibody-epr19812-ab205270
EGR1, rabbit https://www.abcam.com/en-us/products/primary-antibodies/egr1-antibody-epr23981-46-ab300449
RNX1, rabbit https://www.ptglab.com/products/RUNX1-Antibody-25315-1-AP.htm?srsltid=AfmBOooRuq32Amnge4kba96D2QgywHdbscWO9HH4Fq8Z4-eEkOfggw_3

Animals and other research organisms

Policy information about studies involving animals; ARRIVE guidelines recommended for reporting animal research, and Sex and Gender in Research

Laboratory animals

Mouse, C57/bl6, transgenic, male and female, age 8 weeks to 18 months.
Unless otherwise specified, C57BL/6J mice (ordered from Charles River, UK; strain code 632) were used. Other mouse strains includes: cell cycle reporter line R26Fucci2aR (Fucci2a) kindly provided by Ian J. Jackson; PDGFRαEGFP mice (stock #007669, Jackson Laboratory); Sox9flox/flox mice (obtained from MRC-Harwell, on behalf of the European Mouse Mutant Archive (https://www.infrafrontier.eu/)); K14-CreER mice (stock #005107, Jackson laboratory); R26mT-mG mice (mTmG; stock #007676, Jackson Laboratory); Col1a2CreER mice (strain #029567, Jackson Laboratory); R26FlConfetti mice (strain #017492, Jackson laboratory, kindly provided by Hans Clevers); R26nT-nG mice (nTnG, strain #023537, Jackson Laboratory); H2B-EGFP mice (CAG::H2B-EGFP; strain #006069, Jackson Laboratory); NOD scid gamma mice (NSG; NOD.Cg-Prkdcscid Il2rgtm1Wjl/SzJ; strain #005557, Jackson Laboratory). Pdgfra-CreERT mice (stock#018280, Jackson Laboratory).

Mice were bred and maintained under specific-pathogen-free conditions at the Gurdon Institute and The Anne McLaren Building, University of Cambridge. All animals were housed between 20-24°C, 45-65% humidity and a 12-hour light-dark cycle.

Wild animals

No wild animals were used in this study

Reporting on sex

All experiments comprised a mixture of male and female mice with no gender-specific differences observed (unless specified otherwise). For RNA sequencing experiments, only male animals were used in order to avoid cofounding effects due to estrous cycle.

Field-collected samples	No field collected samples were used in this study
Ethics oversight	All experiments were approved by the local ethical review committees of the University of Cambridge and conducted according to Home Office project licenses PPL70/8866 and PP7037913 at the Gurdon Institute and The Anne McLaren Building, University of Cambridge.

Note that full information on the approval of the study protocol must also be provided in the manuscript.

Plants

Seed stocks	*Report on the source of all seed stocks or other plant material used. If applicable, state the seed stock centre and catalogue number. If plant specimens were collected from the field, describe the collection location, date and sampling procedures.*
Novel plant genotypes	*Describe the methods by which all novel plant genotypes were produced. This includes those generated by transgenic approaches, gene editing, chemical/radiation-based mutagenesis and hybridization. For transgenic lines, describe the transformation method, the number of independent lines analyzed and the generation upon which experiments were performed. For gene-edited lines, describe the editor used, the endogenous sequence targeted for editing, the targeting guide RNA sequence (if applicable) and how the editor was applied.*
Authentication	*Describe any authentication procedures for each seed stock used or novel genotype generated. Describe any experiments used to assess the effect of a mutation and, where applicable, how potential secondary effects (e.g. second site T-DNA insertions, mosiacism, off-target gene editing) were examined.*
